# Strong and Flexible Braiding Pattern of Carbon Nanotubes for Composites: Stiff and Robust Structure Active in Composite Materials

**DOI:** 10.3390/ma16041725

**Published:** 2023-02-19

**Authors:** Fumio Ogawa, Fan Liu, Toshiyuki Hashida

**Affiliations:** Fracture and Reliability Research Institute, Tohoku University, 6-6-11, Aza-Aoba Aramaki, Aoba-ku, Sendai-shi 980-8579, Japan

**Keywords:** carbon nanotubes, fabric composites, braiding pattern, natural products, finite element analysis, strength and flexibility

## Abstract

Carbon nanotubes (CNTs) exhibit high strength, Young’s modulus, and flexibility and serve as an ideal reinforcement for composite materials. Owing to their toughness against bending and/or twisting, they are typically used as fabric composites. The conventional multiaxial braiding method lacks tension and resultant strength in the thickness direction. Some braiding patterns are proposed; however, they may have shortcomings in flexibility. Thus, this study proposed three types of braiding pattern for fabrics based on natural products such as spider net and honeycomb, in accordance with thickness-direction strength. The spider-net-based structure included wefts with spaces in the center with overlapping warps. At both sides, the warps crossed and contacted the wefts to impart solidness to the structure and enhance its strength as well as flexural stability. In addition, box-type wefts were proposed by unifying the weft and warps into boxes, which enhanced the stability and flexibility of the framework. Finally, we proposed a structure based on rectangular and hexagonal shapes mimicking the honeycomb. Moreover, finite element calculations were performed to investigate the mechanisms through which the proposed structures garnered strength and deformation ability. The average stress in fabrics becomes smaller than half (43%) when four edges are restrained and sliding is inserted. Under three-dimensional forces, our proposed structures underwent mechanisms of wrapping, warping, sliding and doubling, and partial locking to demonstrate their enhanced mechanical properties. Furthermore, we proposed a hierarchical structure specialized for CNTs, which could facilitate applications in structural components of satellites, wind turbines, and ships. The hierarchical structure utilizing discontinuity and sliding benefits the usage for practical mechanical systems.

## 1. Introduction

**Figure 1:**
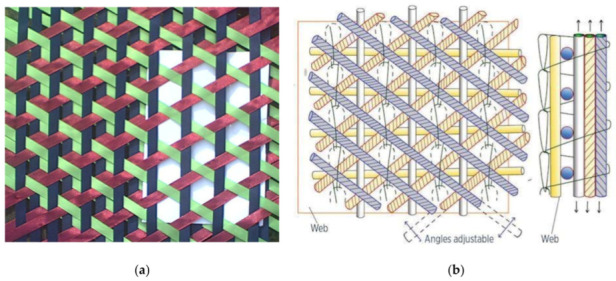
Multiaxial weaving pattern (from web). (**a**) Triaxial weaving; (**b**) multiaxial warp knit pattern.

Recently, composite materials have been used in various fields such as aerospace, the automobile industry, energy production, and sports equipment [1,2,3,4,5] owing to their strength and stability. Fabric composites are widely used because their mechanical and physical properties can be designed depending on their braiding pattern [6,7,8,9,10]. In particular, several types of braiding patterns are used in structures such as ablators in rockets and vibration suppression members in satellites. Furthermore, multiaxial weaving, such as biaxial and triaxial patterns, has been widely used in construction, aerospace applications, and energy production [11,12,13,14]. The representative multiaxial weaving pattern used in composite materials is presented in Figure 1.

In principle, multiaxial weaving utilizes the contact of each web to enhance the stability of the structure, wherein the interaction at the cross point increases the strength of the fabrics. Moreover, the space between the webs can be adjusted to improve the flexibility of the structure (Figure 1a). The multiaxial warp knit pattern proposed by an aerospace engineering corporation is illustrated in Figure 1b, wherein the knitting web enhances the three-dimensional stability of the structure by orienting the sewing knit along the thickness direction.

Generally, multiaxial weaving is advantageous because of its relatively simple structure and stability. They are ideal constituents for the composite materials used in aircrafts and satellites. However, the deficiency of strength along the *z*-direction and the structural weakness caused by the repetitive 2D-weaving pattern form the significant limitations of multiaxial weaving. Moreover, failure may occur at a point located farther from the intersection points of the aggregated webs [15,16]. Thus, developing a three-dimensional braiding pattern exhibiting strength in multiple directions is imminent. The contact of the webs increases the strength, but the sliding motion between them may enhance their flexibility against torsion and impact, which are frequently applied in aerospace (e.g., ablator subjected to thermal loading) and satellite applications.

This study proposes a braiding pattern for carbon nanotubes (CNTs)—a new material exhibiting high strength, Young’s modulus, and electrical conductivity. Complex patterns can be achieved because of their resistance against cutting under bending and/or twisting. In addition, we propose braiding patterns based on natural paradigms (e.g., spider net and honeycomb) and investigate the mechanisms that generate strength and flexibility through finite element analysis (FEA). Specifically, the braiding patterns were developed with reference to the natural systems, i.e., the framework is created with combinations of wefts and warps by utilizing the reaction forces and localized slip.

Finally, the braiding pattern specialized for CNTs was obtained by combining the curled and straight webs with biased repetitions of dense webs. Furthermore, we discuss the similarities between the braided fabrics and a highly stable, sophisticated mechanical board, e.g., wood.

The novelty of this study is the proposal of a newly developed braided pattern, justified numerically using FEA. The insertion of sliding and hierarchical structure leads to the balance of strength and flexibility, evidenced by comparing with other studies in the literature [17,18,19]. Adoption of webs discontinuity also merits the improvement of strength and flexibility.

## 2. Development of Braiding Patterns

### 2.1. Braided Spider Net

Herein, we propose a braided pattern for CNTs based on a typical spider net, which is a natural product with high strength and flexibility. Spider nets exhibit nonlinear stiffening and increased robustness in case of abrupt load variations [20]. Although the contact of the webs increases strength, it can extend the failure strain throughout the entire spider net.

**Figure 2:**
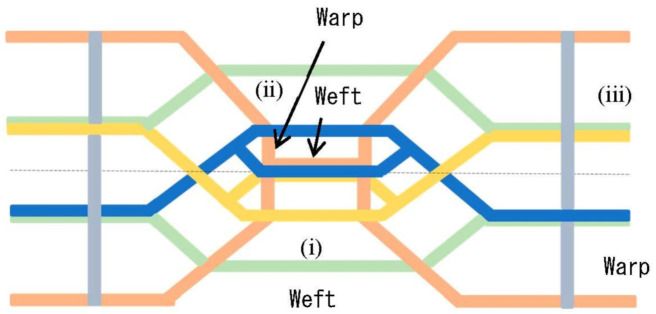
Braiding pattern based on spider net.

The basic braiding pattern is illustrated in Figure 2, wherein the blue and yellow webs denote wefts with space in the center. They intersect the orange warps to enhance sliding and the resultant flexibility. The light-green weft contacts the orange warps to improve stability. In particular, the gray warps overlap three types of wefts (blue, yellow, and light-green webs) and form a zigzag structure that increases the structural strength of the fabrics. To braid this pattern, the following procedure is required: (i) placing wefts in which sliding is enhanced; (ii) sewing warps crossing over wefts; and (iii) placing wefts that contact the warps and create a zigzag pattern at both ends of the braided products. Nonetheless, as the braiding machine might require further development, we aimed to generate a simple braiding pattern.

**Figure 3:**
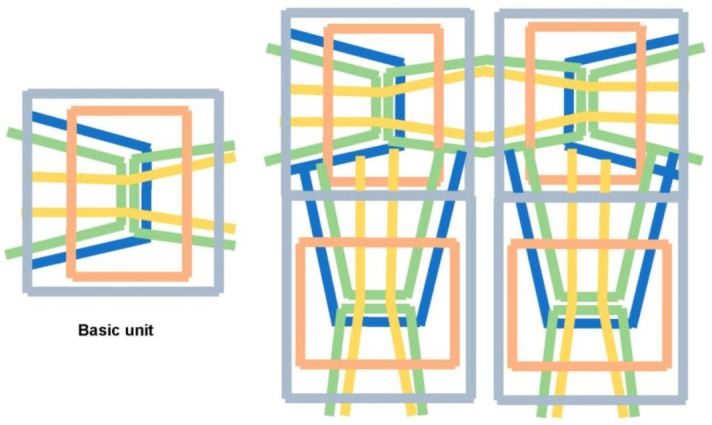
Braiding pattern with wefts and warps unified into boxes and portion of wefts combined outside the boxes enhancing structural stability.

The braiding pattern with the weft and warps unified into a box is displayed in Figure 3. The basic unit is visualized on the left-hand side of Figure 3, which is combined to form the braided structure. The basic units are stacked in the horizontal direction and the wefts are combined to strengthen the stability. Thereafter, the basic unit is rotated in the vertical direction and the green and yellow warps are connected with the blue wefts on the upper side. Although this enhances the structural strength of the fabrics, creating specific free points may provide flexibility to the structures. Moreover, a partly locked sewing machine can be utilized to create a braided pattern that can withstand vibration and impact [21,22]. The development of the braiding machine is reported elsewhere.

### 2.2. Braided Pattern Based on Honeycomb

**Figure 4:**
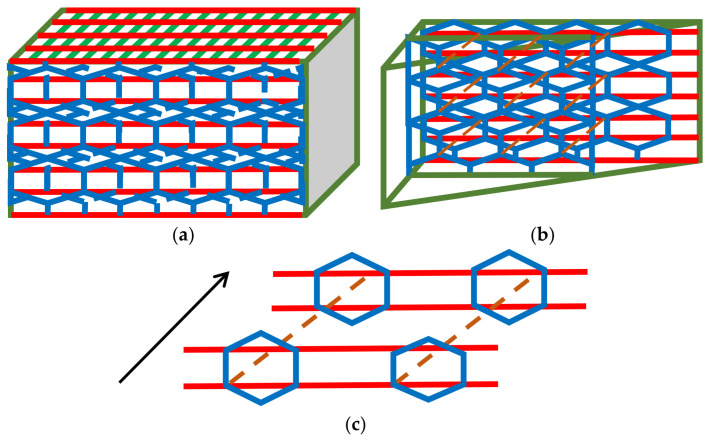
Braiding pattern based on honeycomb: (**a**) front view; (**b**) cross section; and (**c**) spatial relationship of each web.

In this section, we propose a honeycomb-based braiding pattern. Honeycomb is constructed by bees to stock honey and is based on self-organization [23]. The natural hexagonal shape offers resistance against compressive loads and high stability [24]. As reported, the hierarchical hexagonal shape exhibits high stability against shear load. In addition, a structure with high compressive strength has been proposed using curled inlets [25]. Herein, we offer a pattern by placing the wefts in the form of rectangles and surrounding them with hexagonal webs (blue line, Figure 4), which follows the hierarchical structure and enhances stability.

The front view of the proposed braiding pattern is projected in Figure 4a, whereas the cross section from the inclined view is depicted in Figure 4b. The red line corresponds to the continuous wefts directed toward the *x*-direction, the blue lines correspond to the hierarchical hexagonal web, and the dashed orange lines indicate the sewing yarn that improves stability against shear and bending load. The spatial relationship between each web is visualized in Figure 4c; the orange sewing yarn enhances the strength of the fabrics by placing them at the intersection of the weft and hexagonal shape web. When torsional stress is applied, the distance between layers increases; however, the sewing yarn impedes the torsional movement owing to the counterforce. This pattern can be facilely braided by developing a specialized machine [26,27].

## 3. Basic Calculation of Mechanical Properties

**Figure 5:**
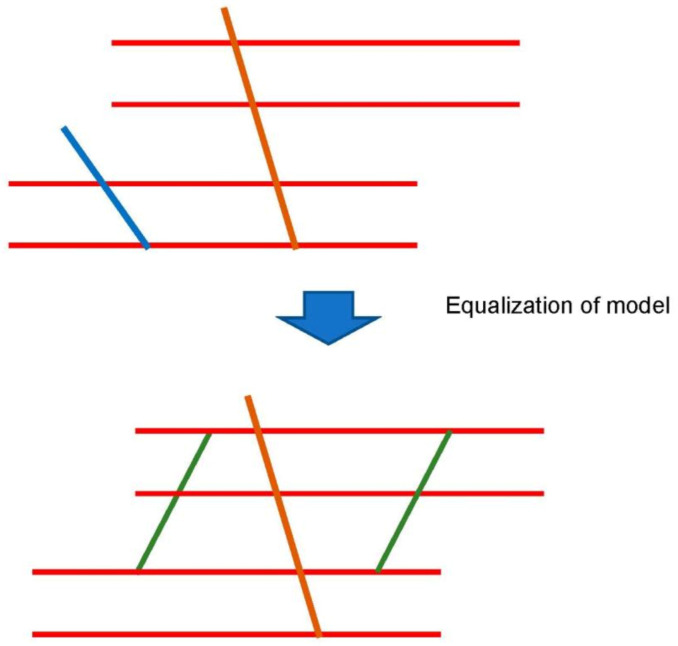
Simplification and equalization of braided pattern into FEA model.

Strength and flexibility are vital characteristics of fabric composites, serving as the reinforcement of composite materials used in various practical applications. Thus, we performed FEA for the model developed based on the braided pattern (Figure 4). The modeling strategy and procedure is described in this section. As depicted in Figure 5, the honeycomb-based web was simplified to the inclined bar (blue line), considering the interaction with sewing yarn (brown line). Blue lines represent the webs that have periodicity. However, it can be simplified to one web, considering the interaction of each web. The brown web represents the effect of counterforce, which impedes the opening of the interlayers.

In this situation, the model was equalized into Figure 4b to calculate the overall stress state inside the fabrics. The commercial FEA code ANSYS Mechanical (Mechanical 2022 R1, 2022, Cybernet, Tokyo, Japan) was used for the calculation. The CNT yarns represented the weft and warp bars of 0.2 mm diameter, which is the minimum size of the modeling tool in ANSYS Mechanical.

**Figure 6:**
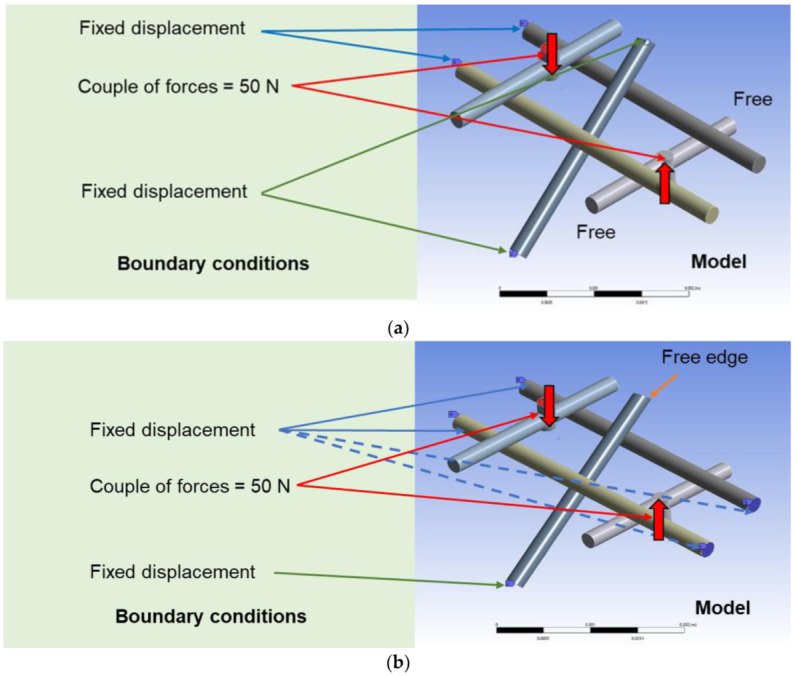

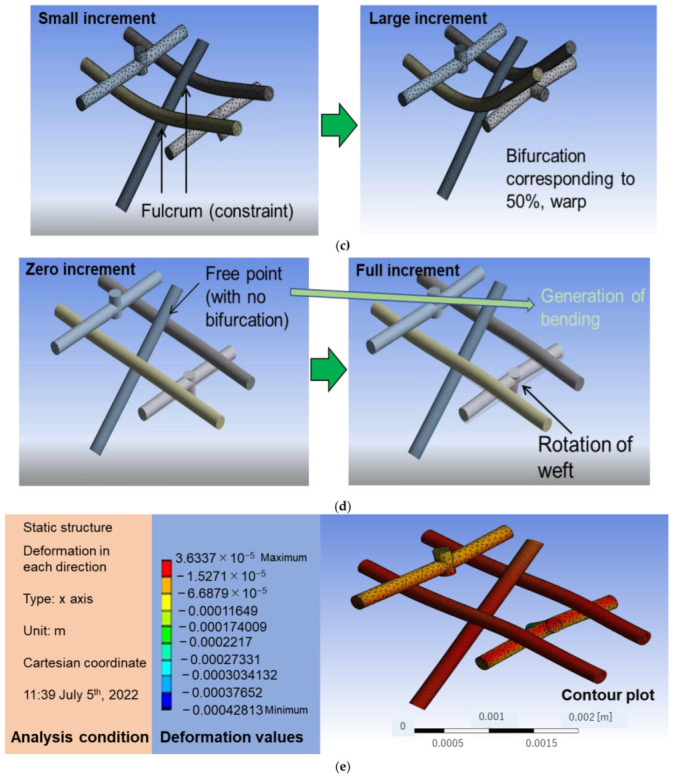
FEA models developed and analysis result. (**a**) FEA model with two constraints on both weft and sewing yarn. (**b**) FEA model with four constraints on weft and one free edge in sewing yarn. (**c**) Deformation mapping of FEA model (A). (**d**) Deformation mapping of FEA model (B). (**e**) Deformation contour of FEA model (B).

The distance between the wefts was set as five times the yarn diameter and that for the warps was set as ten times the yarn diameter. The relationship between each yarn was set to the CONTACT (contact analysis). The most severe constraint was applied as a condition of the contact analysis. In particular, the mesh is generated based on an adaptive method using approximately 70,000 elements in the calculation. The p-method for adaptive mesh (higher order element) was adopted. A large deformation analysis was performed considering deformation under torsion loading. The nobs were placed at the centers of the warps and two forces of 50 N were symmetrically applied. The Young’s modulus of the yarn was set at 200 GPa with a Poisson’s ratio of 0.3. The reason for the adoption of two values are the following: Conventional carbon fiber has a Young’s modulus of approximately 200 GPa and multi-ply twisted CNT yarns have approximately this value. Setting severe conditions leads to the limited analysis of the design stress. The repeated use of CNT yarns increases Poisson’s ratio; therefore, the value of 0.3 was adopted. The parameters and conditions used in FEA analysis are summarized in Table 1. Overall, two types of boundary conditions were adopted. In the first condition, the left-side ends of the wefts as well as both ends of the inclined sewing yarn were fixed in displacement (Figure 6a). In the second type of boundary condition, four ends of the wefts were fixed in displacement. In the case of the sewing yarn, only the lower side was fixed in displacement, whereas the upper side was set to the free edge (Figure 6b). The situation in which torsional stress is applied is assumed; the temperature variation in the mechanical parts and the impact with two contact points associated with tensile stress leads to the generation of torsional load.

The elastic analysis assisted by CONTACT revealed large deformations in each element. Owing to limited computational stability, the calculation was terminated at the point of convergence. However, the accuracy of the analyses was ensured by reviewing the stability (residue) and repeating the calculations. The maximum and minimum stress generated in the model including the average stress are listed in Table 2.

In model (A), large deformations occurred and the average stress was higher because of the large curvature (Figure 6c). Contrarily, in model (B), the deformation was under constraint and the average stress was less than half of that of model (A) (Figure 6d).

Figure 6d depicts that the free edge at the upper side of the sewing yarn leads to generation of bending in the warp in the depth direction and rotation of weft. The contour of deformation is also indicated in Figure 6e. Although the maximum stress in model (A) was notably high, its minimum stress was less than that of model (B). In contrast, the maximum stress in model (B) was less, regardless of the higher minimum stress. These findings indicated that the friction between the yarns reduced the average stress in model (B). Therefore, the free edge can effectively reduce the load and improve fracture resistance.

Owing to the high value of the elastic modulus in this analysis, the maximum stress tended to become impractically large. However, the obtained stress values depend on the elastic modulus of the yarns. Considering that the actual elastic modulus of the single CNT yarn was approximately 30 GPa, the maximum stress increased to ~50 GPa (average stress should be limited to 210 MPa).

One analysis takes a duration of approximately 2.5 h; checking and analyzing the displacement and stress during analysis suggests that some numerical vibration occurs due to the insertion of sliding. However, fixation leads to the convergence of numerical calculation. This suggests some interaction of webs (wrapping, warping, and doubling) takes place in the braided fabric.

**Figure 7:**
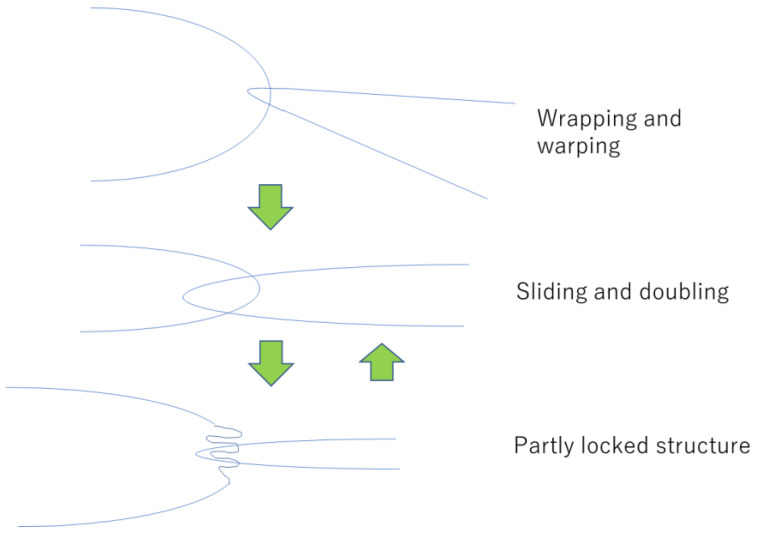
Mechanisms of interaction for each web in braided fabrics.

In this case, we determined that an appropriate constraint adjustment can reduce the load and promote the fracture resistance. In the following section, we propose the mechanism for increasing the toughness against bending and torsion fracture. The interaction mechanism of each web in the braided fabrics is illustrated in Figure 7.

In the first stage, a web overhangs and encloses another web, after which warping occurs. Subsequently, it presumes the original shape under the occurrence of sliding and doubling with the weak interaction between each web. Finally, local unevenness is generated that strengthens the interaction. This state is called a partly locked structure. The intervention by friction and restoration to “sliding and doubling” may reduce the overall stresses. The arrangement of each web in the fabric should be carefully considered to simultaneously improve the strength and avoid failure at lower strains.

## 4. Consideration: Braided Patterns Revisited and Proposal of Mechanisms to Maintain Overall Stability

### 4.1. Braiding Patterns with Inclined Reinforcement Bar Assisted by Diagonal Bridges

Based on the discussed results, we formulated the following strategy to enhance the fabric strength and fracture resistance.

1. Construction of a stiff framework to improve strength.
2. The sliding at the contact point of webs is beneficial to reduce the stresses; however, this is the degree problem.
3. A hierarchical structure is essential to avoid unfavorable overload and enhance flexibility.

**Figure 8:**
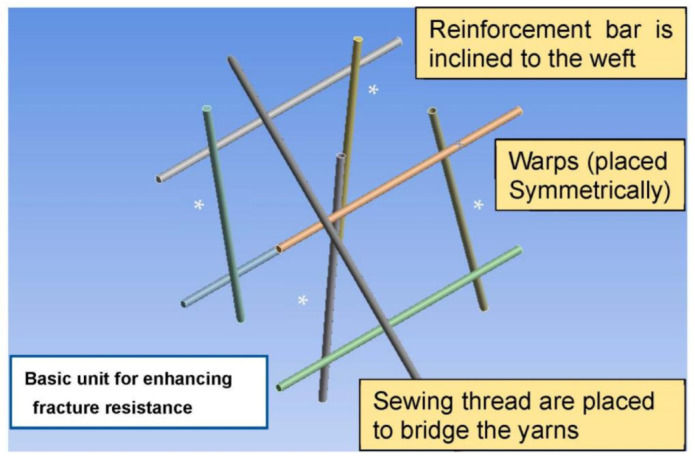
Basic unit of braided fabrics to enhance fracture resistance.

The spider net fulfills the aforementioned aspects of the formulated strategy. Accordingly, the following basic units are proposed for fabricating the braided fabrics (Figure 8). In the model, four wefts are placed in parallel and the inclined reinforcement bars (indicated by asterisks (*)) are set to contact with the wefts. The sewing yarn is positioned to bridge the wefts.

**Figure 9:**
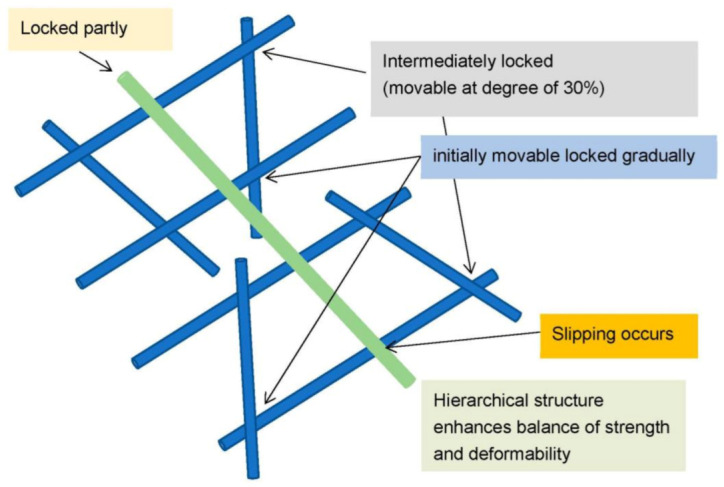
Operational availability (movable degree) of contact points of webs.

The relationship between the webs is presented in Figure 8. The top- and bottom-right intersection points were partly locked but movable at a degree of 30%. The lower right and its symmetry point were set as initially movable but gradually locked. The slipping in the inclined sewing yarn occurred at the lower side, whereas the upper side was partly locked. The hierarchical structure was constructed to achieve an excellent balance between strength and flexibility. Herein, we discuss the variations in the local moments at the contact point. In Figure 9, the force balance is indicated by arrows; the force is transferred from the intermediate locked point to the initially movable (partly free) point, and subsequently, it is transferred to the symmetrical edge. Ultimately, the force is transferred to the sewing thread and the bottom portion is subjected to a large load. Similar phenomena have been reported in ref. [28]. The sliding enhances flexibility and gradually transfers the load.

**Figure 10:**
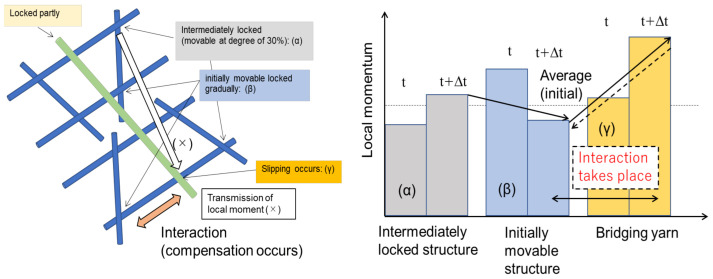
Variation in the local moment at each point in the model unit.

The variations in the local moments at each point are charted in Figure 10, wherein the grey bar indicates the local moments at the intermediately locked structure (α) and the light blue bar represents that at the initially movable point (β). As signified by the orange bar (γ), the moment increased considerably at the bridging yarn. The initially movable point experienced a marginally more decisive moment than the intermediate locked structure at the initial moment; however, it decreases because the overall stress was rapidly transferred to the free point. Subsequently, the interaction of two points occurs; the moment at the initial movable point increases again. The balance determines local stresses.

The theoretical evidences have been presented in refs. [29,30,31].

### 4.2. Ultimate System Comprising CNTs Available in Composites and Engineering Belts

**Figure 11:**
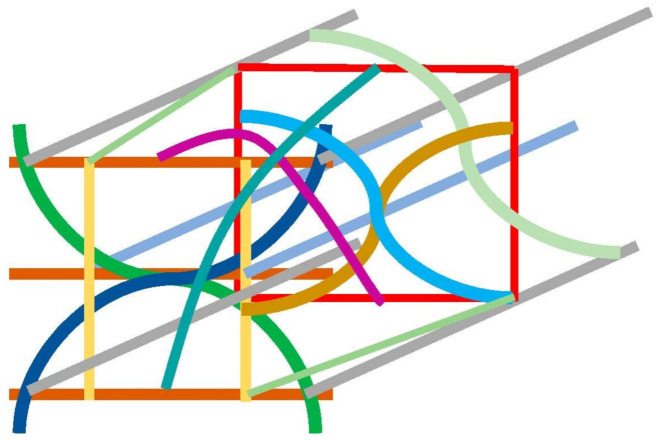
Braided pattern specialized for CNTs: curled and straight CNT webs are combined and the density slope was utilized to enhance overall stability.

**Figure 12:**
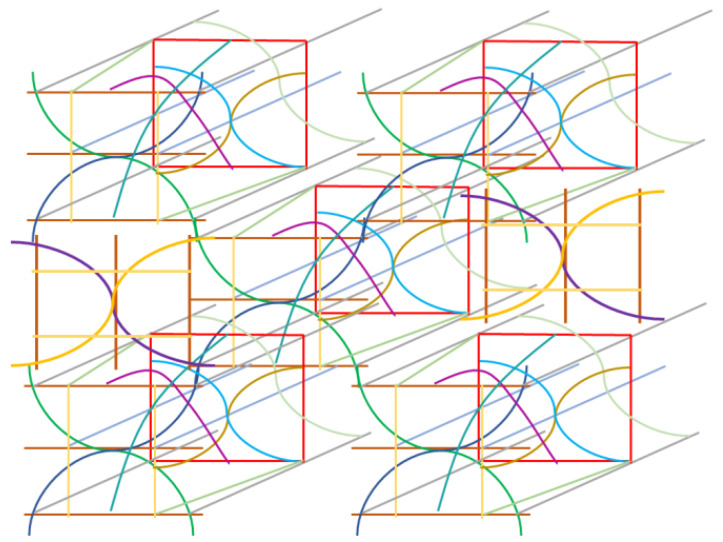
Three-dimensional “assembled” braiding pattern for CNT fabrics.

Finally, we propose a three-dimensional braiding pattern specialized for CNTs. Based on the discussed results, constructing the framework and enhancing the sliding motion between the webs are crucial for obtaining strong and flexible braided fabrics. The ultimate braiding pattern for CNTs is displayed in Figure 11, wherein the blue and green lines represent the curled CNT webs and the gray and light-blue lines indicate the straight CNT webs. In the vertical direction, the yellow lines were arranged as reinforcing webs. In the depth direction, the boxes composed of red webs were connected with the front side by the purple and dark-green web to improve the mechanical strength of the fabric. Inside the red boxes, the overlapping of the three curled CNT webs increased the density of the webs. The density gradient was set to enhance the overall stability. The fundamental unit used in generating the fabrics is depicted in Figure 11, and the overall assembled braided pattern is presented in Figure 12.

The structure is the intermediate presence of the spider net and honeycomb structures (which utilizes sliding, continuity, discontinuity, and periodicity).

In the following, studies from the literature regarding the braided composites are mentioned. Saleh et al. [17] proposed braided structures for carbon fiber; three types were proposed: orthogonal interlock (ORT), layer-to-layer (LTL), and AI (angle interlock). Bearing strength tests of the three types were performed. They reported that ORT is superior in ductility; on the contrary, LTL has merit in load-bearing capacity. The above proposed structure (Figure 11) represents a mixture of ORT and AI and in part it corresponds to LTL in a certain direction. It represents the generation of higher flexibility in accordance with high strength. The proposed structure may have intermediate properties proposed by the literature studies. The tensile strength of multiple twisting yarns fabricated in our laboratory reaches approximately 600 MPa (although omitted here and planned to be reported in the near future); therefore, tensile a strength over 400 MPa is expected for composites. Moreover, it has merit in the balance of adaptive forces, leading to the generation of ductility.

The hierarchical structure (Figure 11) is illustrated in Figure 12, wherein the blue and green webs are arranged continuously to enhance sliding. In addition, the straight webs are placed continuously and the red boxes do not contact each other. However, such an arrangement can ensure the mechanical stability because the repeated pattern of the curled CNT webs inside boxes enhances the mechanical strength of the framework. Moreover, the vacant space can be filled by rotating a basic unit by 90° to achieve structural continuity. The discontinuity at the purple and dark-green webs inhibits the stress concentration and distributes phase strain. The structure deforms partly, and flexibility is secured.

Herein, we discuss the analogy of the proposed pattern with respect to a continuous structural board material, e.g., wood. The braided pattern exhibits structural strength against tensile load and is stable against torsional load. Strong interactions can be generated between the webs and the matrix if this system is impregnated with resin or ceramics such as a carbon matrix. In particular, bridging the webs improves strength and sliding enhances the flexibility. Upon impregnation, the “pore” and “core” might be generated. Although a “pore” corresponds to voids, it can be filled by the matrix as it turns to “core.” In the proposed braiding pattern, continuous and discontinuous webs were combined to generate a strong and flexible structure. In the conventional mechanical board material, the stress is continuously transferred to yield stiffness and fracture resistance. In the proposed system, the load is transferred intermittently after impregnation. However, the presence of matrix supports the load transfer and steadily distributes the load. The characteristic of the proposed pattern (Figure 11 and Figure 12) is that the load that cannot be transferred through local matrix is borne by the neighboring webs; it also exhibits strong resistance to high bending and torsional load (tensile strength can be appropriately designed). The composites utilizing this pattern can be employed in wind turbines, aerospace applications, satellites, and the structural components of ships. Patterns resembling the braided pattern proposed herein have been reported earlier [32,33,34,35]. However, the originality of this study is in utilizing both the stiffness and sliding characteristics associated with the hierarchical structure. Furthermore, the proposed pattern can be utilized in electrically and thermally conductive materials as well as metal and ceramic matrix composites. The material production following this proposal will reported in a follow-up study.

## 5. Conclusions

1. This study proposed three types of braiding patterns based on spider net and honeycomb. The first pattern combines sliding and tight-yet-flexible sewing between the wefts and warps to provide both strength and flexibility. The second pattern combines the wefts and warps into boxes to balance strength and flexibility by utilizing the continuity and discontinuity. The final pattern was generated through the combination of rectangular wefts and hexagonal webs. The wefts were surrounded by hierarchical hexagonal webs, with variations in their positions in the depth direction. This results in the stress distribution and resilience to fracture at low strains.
2. The basic FEA analysis was performed for the honeycomb pattern, which elucidated that the load borne by the webs and the partly generated slippage contributed to the reduction in overall stresses. The constraint between the webs does not necessarily increase the stress in CNT webs and utilizing their interaction can effectively yield both strength and fracture resilience with the generation of flexibility. Thus, wrapping and warping, sliding and doubling, and partly locked structures have been proposed as a mechanism for generation of strength and flexibility. The recovery of structures from the interaction to the independent state is vital to generate fracture resistance with low strains.
3. We propose the finalized structure specialized for CNTs. The combination of curled CNTs and straight CNTs can transfer the load associated with tension and sagging. The hierarchical pattern in which the braiding density is spatially altered is unique and can generate strong and resilient structural components. Compared with conventional braiding patterns, a balance of strength and flexibility can be expected in our proposed braiding pattern. Furthermore, we discussed its similarities and dissimilarities with high-stiffness structural board materials.

## Figures and Tables

**Table 1:** Parameters and conditions used in the FEA analysis.

Parameters	Value	Mesh Type	Analysis Method
Young’s modulus	200 GPa (Assuming multiply twisted yarns)	Adaptive mesh (P-method) Approximately 70,000 elements	Contact (Severe constraint) Large deformation (Compensate)
Poisson’s ratio	0.3		

**Table 2:** Maximum, minimum, and average stresses in the FEA model.

Model	Maximum Stress (GPa)	Minimum Stress (Pa)	Average Stress (MPa)
(A)	392	39.7	2402
(B)	255	3012	1044

## Data Availability

Basic data can be obtained from the first (corresponding) author.
